# Coltiviruses and Seadornaviruses in North America, Europe, and Asia

**DOI:** 10.3201/eid1111.050868

**Published:** 2005-11

**Authors:** Houssam Attoui, Fauziah Mohd Jaafar, Philippe de Micco, Xavier de Lamballerie

**Affiliations:** * Université de la Méditerranée, Marseille, France

**Keywords:** Coltivirus, seadornavirus, dsRNA viruses, Colorado tick fever, Banna virus, encephalitis, vectorborne virus, synopsis

## Abstract

Coltiviruses are tickborne viruses of the genus *Coltivirus*. The type species, Colorado tick fever virus (from North America), has been isolated from patients with flulike syndromes, meningitis, encephalitis, and other severe complications. Another coltivirus, Eyach virus, has been isolated from ticks in France and Germany and incriminated in febrile illnesses and neurologic syndromes. Seadornaviruses are endemic in Southeast Asia, particularly Indonesia and China. The prototype virus of the genus, Banna virus (BAV), has been isolated from many mosquito species, humans with encephalitis, pigs, and cattle. Two other seadornaviruses, Kadipiro and Liao Ning, were isolated only from mosquitoes. The epidemiology of seadornaviruses remains poorly documented. Evidence suggests that BAV is responsible for encephalitis in humans. Infection with BAV may be underreported because it circulates in regions with a high incidence of Japanese encephalitis and could be misdiagnosed as this disease.

## Abstract 2

Neurotropic virus disease is often misdiagnosed as Japanese encephalitis.

Vertebrate viruses belonging to the family *Reoviridae* and having 12-segmented dsRNA genomes at one time were classified in the genus *Coltivirus* (Colorado tick virus [CTFV]). At the time the genus was created, it included tickborne and mosquitoborne viruses. Presently, the genus *Coltivirus* contains only CTFV, California hare coltivirus (CTFV-Ca), which is considered a serotype of CTFV, Eyach virus (EYAV), which is distinct from CTFV, and Salmon River virus (SRV), which may be a serotype of both CTFV and CTFV-Ca. Alternatively, analysis of sequence data and antigenic properties of the mosquitoborne viruses led to their reassignment to a new genus designated *Seadornavirus* (Southeast Asian dodeca RNA virus) (*1**,**2*).

## Coltiviruses

### Historical Aspects and Epidemiology

Tickborne vertebrate viruses include CTFV isolated from humans and ticks, EYAV isolated from ticks, CTFV-Ca isolated from a hare (*Lepus californicus*, black-tailed jackrabbit) in northern California, and SRV isolated from a person in Idaho. Many mosquitoborne viruses were also considered tentative species in this genus, including Banna virus (BAV) from humans. The tickborne viruses in this genus have many distinctive features and sequence data have led to a reevaluation of their taxonomic status.

Coltiviruses have been isolated from ticks of the family *Ixodidae* and from rodents and humans. CTFV is endemic in northwestern North America, where it causes CTF, a human disease initially confused with a mild form of Rocky Mountain spotted fever, which is caused by *Rickettsia rickettsii*. The causative agent was isolated by Florio and others (*3*) in 1946 from human serum by injection into adult hamsters. The virus was adapted to egg and mice, and suckling mice became the routine isolation system for CTFV (*3**–**5*).

CTF is found in the Rocky Mountain region of the United States and in Canada. The virus distribution closely matches that of its vector *Dermacentor andersoni*. CTFV-Ca, identified as strain S6-14-03, was isolated from the blood of the white hare, *L. californicus*, in California (outside the range of *D. andersoni*) (*6*). Antibodies to CTFV antigen have been detected in sera of humans in South Korea (C. Calisher, pers. comm.). SRV was isolated from a patient with moderately severe CTF-like illness in Idaho.

EYAV was isolated in Europe in 1976 from *Ixodes ricinus* ticks (EYAV-Gr) and in 1981 from *I. ricinus* (EYAV-Fr578) and *I. ventalloi* (EYAV-Fr577) (*7*). Its antigenic relationship to CTFV was established by a complement-fixation assay (*7*). Genome sequence analysis showed that CTFV and EYAV are closely related (*8*) and confirmed the antigenic observations.

Serologic surveys in France identified antibodies to EYAV in 1.35% of animals, including the European rabbit (*Oryctolagus cunniculus*), mice, mountain goats, domestic goats, sheep, and deer (*9*). The presence of EYAV was suspected in Europe because anti-EYAV antibodies were detected in patients with neurologic disorders. However, the natural cycle of the virus is still unclear, and whether it circulates continuously in Europe is not known, although the rabbit *O. cunniculus* is suspected of being main host (*6**,**9*). In 2003, the virus was reisolated from *I. ricinus* ticks in Germany (*10*).

### Vectors, Host Range, and Transmission

Ticks are the principal vectors of coltiviruses. CTFV is transmitted by the wood tick *D. andersoni*, but other ticks such as *D. occidentalis*, *D. albopictus*, *D. arumapertus*, *Haemaphysalis leporispalustris*, *Otobius lagophilus*, *Ixodes sculptus*, and *I. spinipalpis* are also infected with the virus. EYAV has been isolated from *I. ricinus* and *I. ventalloi* (*7*). Ticks become infected through blood meals from an infected vertebrate host. CTFV is transmitted transstadially, but not transovarially. Infected larvae and nymphs can hibernate, and the nymphal and adult ticks become persistently infected. In certain rodents viremia can persist for >5 months. These ticks and rodents may provide hosts by which the virus could overwinter. The prevalence of viremia in rodents in a virus-endemic area ranges from 3.5% to 25%, and the prevalence in ticks ranges from 10% to 25% (*7*). CTFV has a wide host range that includes ground squirrels, chipmunks, wild mice, wood rats, wild rabbits and hares, porcupines, marmots, deer, elk, sheep, and coyotes.

Person-to-person transmission of CTFV can occur by blood transfusions (*11*). This virus is included on the list of agents screened before bone marrow transplantation in the United States (http://www.guideline.gov/summary/summary.aspx?doc_id=2573&nbr=1799&string=pertussis). Prolonged viremia observed in humans and rodents is due to the intraerythrocytic location of virions, which protects them from immune clearance (*12**–**14*).

### Properties, Genome, and Replication

**Figure 1:**
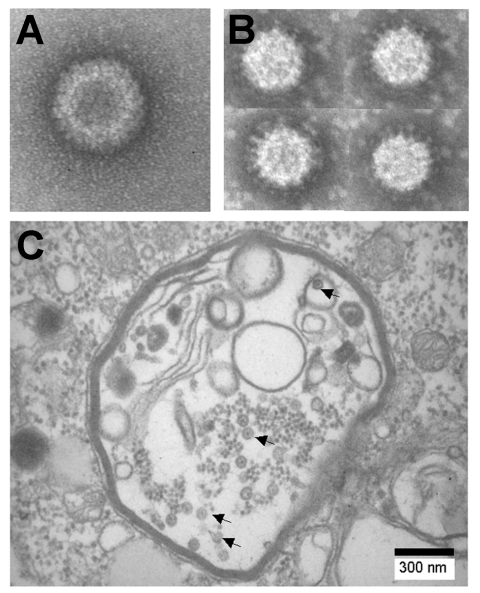
Negative contrast electron micrographs of A) Colorado tick fever virus and B) Banna virus (BAV). C) Thin section of BAV-infected C6/36 cells showing viral particles (arrows) in vacuolelike structures.

Coltivirus particles are ≈80 nm in diameter and have a core ≈50 nm in diameter. Electron microscopic studies (*8**,**15*) have shown particles with a relatively smooth surface capsomeric structure and icosahedral symmetry (Figure 1A). Most viral particles are nonenveloped, but a few acquire an envelope structure during passage through the endoplasmic reticulum (*8**,**15*). The buoyant density of CTFV in CsCl is 1.36–1.38 g/cm^3^. The virus is stable between pH 7.0 and 8.0 but loses infectivity at pH 3.0. CTFV can be stored at 4°C for 2–3 months in 50% fetal calf serum, 0.2 mmol/L Tris-HC1, pH 7.8, or for years at –80°C. Upon heating to 55°C, CTFV loses its infectivity. The virus is stable when treated with nonionic detergents (such as Tween 20) or with organic solvents (such as Freon 113 or its ozone-friendly substitute Vertrel XF), but viral infectivity is abolished by treatment with sodium deoxycholate or sodium dodecyl sulfate (*16**,**17*).

**Figure 2:**
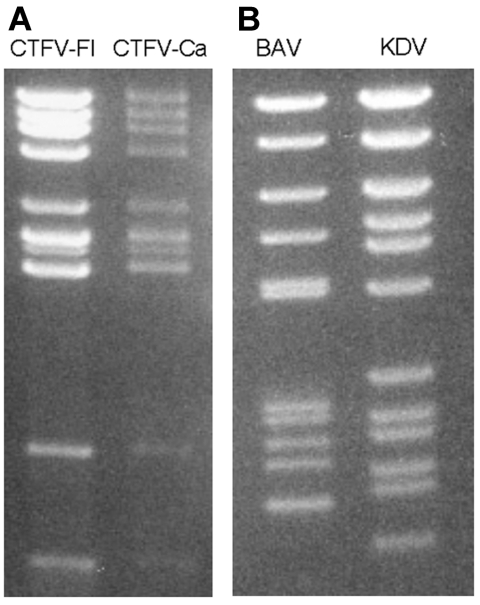
Electropherotypes of coltiviruses and seadornaviruses on 1% agarose gels. A) Colorado tick fever virus (CTFV-Fl) and California hare coltivirus (CTFV-Ca). B) Banna virus (BAV) and Kadipiro virus (KDV).

The genome consists of 12 dsRNA segments designated Seg-1 to Seg-12 in order of reduced molecular weight as observed during agarose and polyacrylamide gel electrophoresis. The genome contains ≈29,000 bp and segment size ranges from 675 bp to 4,350 bp (*18**,**19*). The genomic dsRNA of CTFV has an electropherotype (Figure 2A) similar to that of CTFV-Ca. CTFV produces a cytopathic effect (CPE) in mammalian cells, including human carcinoma cells, monkey kidney cells (buffalo green monkey [BGM] and Vero), hamster kidney cells (BHK-21), and mouse fibroblasts (L-929). Cells infected with CTFV develop granular matrices that contain viruslike particles in the cytoplasm. These structures are similar to viral inclusion bodies produced during orbivirus infections (*15*). In addition, bundles of filaments (tubules) characterized by cross-striations and kinky threads are found in the cytoplasm and, in some cases, in the nucleus of infected cells (*8**,**15**,**17*). These tubules may also be comparable to those found in orbivirus-infected cells. More than 90% of virus particles remain associated with debris after cell disruption.

**Figure 3:**
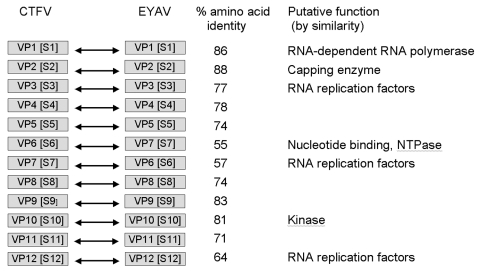
Comparison of nucleotide and amino acid sequences of genome segments of viral proteins (VP) and dsRNA segments (S) of Colorado tick fever virus (CTFV) and Eyach virus (EYAV). NTP, nucleoside triphosphatase.

Sequence analysis of coltivirus genomes has shown that segment 6 of viral protein 6 (VP6) of CTFV is homologous to segment 7 (VP7) of EYAV (Figure 3). The amino acid sequence (residues 370–490) of VP7 of EYAV showed 50% similarity to the sarcolemmal-associated protein of the European rabbit *O. cunniculus*, which may be the major host of EYAV. By comparison, VP6 of CTFV showed no similarity with this rabbit protein (*8*), which may be the result of insertion of a sequence encoding a lagomorph protein into segment 7 of EYAV. Reading through a stop codon, which is common in retroviruses and alphaviruses, was reported in coltiviruses, particularly in segment 9, where long and short proteins are produced from a single open reading frame (*20*).

### Virus Relationships

Antigenic variation between CTFV strains is low, especially between strains from humans (*21*). Distinct CTFV serotypes are difficult to define (*22*), and immunity to reinfection has been observed (*23*). CTFV from North America and EYAV from Europe show little cross-reactivity in neutralization assays. CTFV-Ca cross-reacted with CTFV, but not with EYAV, and may be a serotype of CTFV. Two species of coltiviruses have been identified: CTFV, with 2 serotypes represented by CTFV-F1 and CTFV-Ca, and EYAV. Overall identities between nucleotide sequences from segments 9, 10, 11, and 12 of CTFV strains range from 90% to 100%: 97%–100% for segment 9, 96%–99% for segment 10, 90%–94% for segment 11, and 94%–96% for segment 12. The degree of identity between nucleotide sequences of segments 1–12 of CTFV and EYAV isolates ranges from 55% to 88% (*19*).

The genome of CTFV contains 29,174 nucleotides, and that of EYAV contains 29,210 nucleotides (*18*). All 12 segments of CTFV and EYAV have conserved sequences that are located at their termini. The motifs 5´-G/CACAUUUG-3´ and 5´-UGCAGUG/C-3´ are found in the 5´ and 3´ noncoding regions of CTFV, respectively, and the motifs 5´-GACAA/UUU-3´ and 5´-UGC/UAGUC-3´ are found in these noncoding regions in EYAV. The 5´ and 3´ terminal trinucleotides of all segments in both viruses are inverted complements (*8*).

Genome characterization has helped shed light on the origin of EYAV. Genetic findings support the hypothesis that EYAV was derived from a CTFV-like ancestor virus that was introduced into Europe through Asia when lagomorph ancestors migrated from North America 5–50 million years ago (*8*). The antigenic and genetic relationships between CTFV and EYAV are further corroborated by their identical morphologic features, as analyzed by electron microscopy (*8*).

### Clinical Features

Infection of humans with CTFV is characterized by abrupt onset of fever, chills, headache, retroorbital pain, photophobia, myalgia, abdominal pain, and generalized malaise. Diphasic or triphasic febrile patterns have been observed, usually lasting for 5 to 10 days. Severe forms of the disease that involve infection of the central nervous system (CNS) or hemorrhagic fever, pericarditis, myocarditis, and orchitis have been rarely observed, mainly in children. Severity is sufficient to result in hospitalization of ≈20% of patients. Offspring of mice experimentally infected with CTFV showed teratogenic effects (*24*). Mother-to-infant transmission has been reported in pregnant women.

The incidence of complications in different reports of infection with CTFV has been reported as <7% (23–26). CTFV causes leukopenia (65% of infected humans), with mean leukocyte counts of 900/μL to 3,900/μL, and thrombocytopenia, with platelet counts of 20,000/μL to 95,000/μL (*27*). Patients with neurologic disorders (meningitis, meningoencephalitis, encephalitis) show lymphocyte infiltration of cerebrospinal fluid (CSF), and the virus has been isolated from CSF.

CTFV infections have been confused with other tickborne diseases such as Rocky Mountain spotted fever (a rickettsial disease), tularemia, relapsing fever, and Lyme disease. However, rash and leukocytosis distinguish Rocky Mountain spotted fever from CTF. Signs in the CNS confuse CTF with other causes of viral meningitis and encephalitis, including St. Louis encephalitis virus, Western equine encephalitis virus, and enteroviruses.

CTFV can be isolated from blood because it is present in circulating erythrocytes for as long as 4 months (*28*), and it infects the hematopoietic progenitor cells and remains sheltered in erythrocytes after maturation. Intracerebral injection of blood into suckling mice is considered the most sensitive isolation system. Reverse transcription–polymerase chain reaction (RT-PCR) was developed for diagnosis of CTFV infection, and this method detected human and tick virus isolates >3 days postinfection in experimentally infected mice (*29*). As little as 1 genome could be detected by using a PCR assay (*29*).

Serologic diagnostic methods based on CTFV-infected cell cultures have also been developed (*29*). These include 1) a complement-fixation test that is relatively insensitive because in 25% of patients complement-fixing antibodies are not detected and in 75% of patients these antibodies appear late after infection; 2) a seroneutralization assay for detection neutralizing antibodies that appear 14–21 days after onset of disease; 3) an immunofluorescence assay that uses CTFV-infected BHK-21 or Vero cells and is an easy and rapid test for detecting anti-CTFV antibodies; and 4) an enzyme-linked immunosorbent assay (ELISA) for immunoglobulin M (IgM) and IgG that appear concurrently or a few days after neutralizing antibodies (peak 30–40 days after infection), However, IgM titers decrease sharply after day 45. An ELISA based on recombinant VP7 and a Western blot based on synthetic VP12 showed good sensitivity in detecting antibodies to CTFV (*29**,**30*).

A complement fixation assay has been developed for detection of EYAV (*6*). It detected anti-EYAV antibodies in 158 Czechoslovakian patients with encephalitis in whom tickborne encephalitis had been diagnosed. This population was also tested for tickborne encephalitis viruses (Kemerovo, Lipovnik, or Tribec) and antibodies to EYAV. Seventeen serum specimens (11%) had only anti-EYAV antibodies. The same test identified anti-EYAV antibodies in 8 (17%) of 47 patients with polyradiculoneuritis and meningopolyneuritis (*31*). Recently, an ELISA based on recombinant VP6 of EYAV was developed. This test selectively identified anti-EYAV antibodies (*32*). An RT-PCR assay based on the sequence of genome segment 12 has also been developed for specific detection of EYAV (*29*).

## Seadornaviruses

### Historical Aspects and Epidemiology

BAV is the type species of the genus *Seadornavirus* (*19*) that includes Kadipiro virus (KDV) and Liao Ning virus (LNV). Vectors for this genus include *Anopheles*, *Culex*, and *Aedes* mosquitoes. These viruses are endemic in Southeast Asia, particularly Indonesia and China (*2*). BAV was first isolated in 1987 from CSF (2 isolates) and sera (25 isolates) of patients with encephalitis in southern China (Yunnan Province). Numerous isolates were also obtained from other patients with encephalitis (*33**,**34*). A virus described as an isolate of BAV was identified in western China (Xinjiang Province) from patients with fever and flulike manifestations (*35*). Virus isolates from pigs and cattle with genomes having the same electropherotype as BAV were also reported (*34*). The isolation from mosquitoes of 12 segmented dsRNA viruses antigenically related to BAV has been reported in various provinces of China, including Beijing, Gansu, Hainan, Henan, and Shanshi, and in central Java, Indonesia. BAV is now classified as a biosafety level 3 arboviral agent (http://www.cdc.gov/od/ohs/biosfty/bmbl4/bmbl4s74.htm).

### Vectors, Host Range, and Transmission

Seadornaviruses have been isolated from *Culex vishnui*, *Cx. fuscocephalus*, *Anopheles vagus*, *An. aconitus*, *An. subpictus*, and *Aedes dorsalis*. BAV, KDV, and LNV are found in tropical and subtropical regions where other mosquitoborne viral diseases, especially JE and dengue, are endemic. Several cases of encephalitis in China have been diagnosed as Japanese encephalitis (JE) without any detection of JEV or specific anti-JEV antibodies. Recently, 89 paired serum samples from these patients were tested by ELISA for anti-BAV IgG antibodies. At least a 4-fold (up to 16-fold) increase in IgG antibodies was observed in 7 cases. An additional 1,141 serum specimens of patients from a large number of health institutes in China, who supposedly had JE or viral encephalitis, were tested for anti-BAV IgM antibodies; 130 samples were positive (*34*).

Seadornaviruses were shown to replicate in adult laboratory mice and were detected in infected mouse blood at 3 days postinfection until day 5 postinfection (*36*). To date, BAV has been isolated only from humans, and KDV and LNV have been isolated only from mosquitoes.

### Properties, Genome, and Replication

Seadornavirus has 7 structural proteins, 5 of which are present in the core (*37*). The viruses are icosahedral with a diameter of 60–70 nm, and the core has a diameter of ≈50 nm. The surface of virus has spikes (Figure 1B) that are similar to those of rotaviruses. The viruses are stable at pH 7.0, and acidity decreases their infectivity (which is lost at pH 3.0). Purified virus can be stored at 4°C or for long periods at –80°C. Viral infectivity is decreased considerably upon heating to 55°C. Organic solvents such as Freon 113 or Vertrel XF can be used for purification of viral particles from cell lysates and do not affect infectivity.

The seadornavirus genome consists of 12 segments of dsRNA known as Seg-1 to Seg-12 in order of decreasing molecular mass observed by gel electrophoresis (Figure 2B). The genome of BAV or KDV is ≈21,000 bp, and the segment length ranges between 862 bp and 3,747 bp (*2*). During replication, viruses are found in the cell cytoplasm within vacuolelike structures (Figure 1C) that are believed to be involved in morphogenesis (37).

Seadornavirus isolates replicate in various mosquitoes cell lines such as C6/36 and AA23 (both from *Ae. albopictus*), A20 (*Ae. aegypti*), and Aw-albus (*Ae. W. albus*). More than 40% of the virus particles are released into the culture medium before cell death and massive CPE (fusiform cells). Infected cells are not lysed, and the virus leaves cells by budding, thus acquiring a temporary envelope (*37*). Late infection results in cell lysis from cell death. Intracellular radiolabeling of viral polypeptides has shown termination of host cell protein synthesis (*37*).

In addition to its capacity to replicate in a large number of mosquito cell lines, LNV is the only seadornavirus that replicates in a variety of transformed or primary mammalian cell lines such as Hep-2 (human carcinoma cells), BGM, Vero, BHK-21, L-929, and MRC-5 (human lung fibroblasts). Infection with LNV results in a massive lytic effect and also kills adult mice.

### Relationships among Seadornaviruses and other *Reoviridae*

Antigenic relationships between seadornaviruses were investigated by using mouse immune sera. BAV from southern China and Indonesia and KDV from Indonesia are classified as distinct species (*1*) and show no cross-reactivity in neutralization tests. These viruses are also antigenically distinct from LNV. Antigenic variations were observed in many isolates from China that showed cross-reactivity with BAV.

Amino acid sequence analysis identified BAV, KDV, and LNV as 3 distinct species (*2*). Identities between homologous proteins ranged from 24% to 42%, with the highest value found in the polymerase gene. Analysis of different BAV isolates has shown the existence of 2 genotypes identified as genotype A (represented by isolates BAV-Ch [China] and BAV-In6423 [Indonesia]) and genotype B (represented by isolates BAV-In6969 and BAV-In7043 [Indonesia]). This grouping is based on sequences of segments 7 and 9: amino acid sequences of segment 7 show an identity of 72% between the 2 genotypes, while those of segments 9 show an identity of 41%. All other proteins among BAV isolates have identities ranging from 83% to 100% (*1**,**19*). Based on a seroneutralization assay, these 2 genotypes were found to represent 2 serotypes of BAV (*38*).

**Figure 4:**
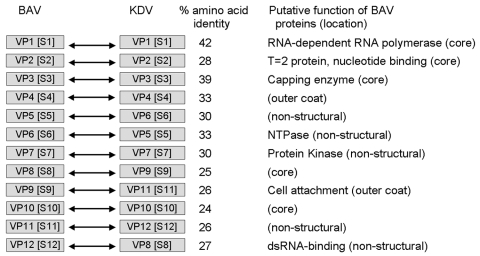
Comparison of nucleotide and amino acid sequences of genome segments of viral proteins (VP) and dsRNA segments (S) of Banna virus (BAV) and Kadipiro virus (KDV). NTP, nucleoside triphosphatase.

**Figure 5:**
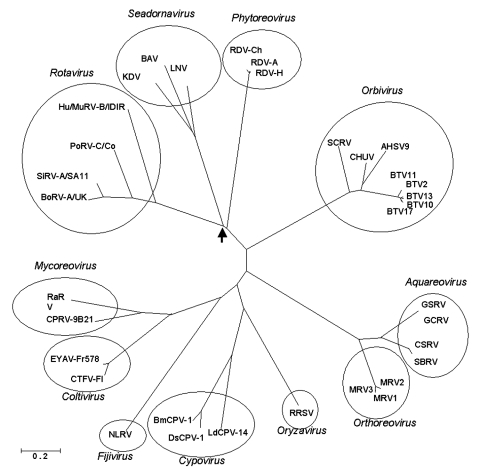
Phylogenetic comparison of the viral polymerase protein sequences of viruses of the family Reoviridae, including seadornaviruses and coltiviruses. Accession numbers and further details of the sequences and viruses are available in the report by Attoui et al. (*8*). Analysis shows coltiviruses and seadornaviruses as 2 distinct phylogenetic groups and that seadornaviruses share a common phylogenetic origin with rotaviruses (arrow). KDV, Kadipiro virus; BAV, Banna virus; LNV, Liao Ning virus; RDV, Rice dwarf virus; RDV-Ch, RDV Chinese isolate; SCRV, St. Croix River virus; AHSV, African horse sickness virus; CHUV, Chuzan virus; BTV, Blue tongue virus; GSRV, Golden shiner reovirus; GCRV, Grass carp reovirus; CSRV, Chum salmon reovirus; SBRV, Striped bass reovirus; MRV, Mammalian orthoreovirus; RRSV, Rice ragged stunt virus; BmCPV, Bombyx mori cytoplasmic polyhedrosis virus; DsCPV, Dendrolimus spectabilis cytoplasmic polyhedrosis virus; LdCPV, Lymantria dispar cytoplasmic polyhedrosis virus; NLRV, Nilaparvata luguens reovirus; CTFV, Colorado tick fever virus; EYAV, Eyach virus; CPRV, Cryphonectria parasitica reovirus; RaRv, Rotavirus A; BoRV, Bovine rotavirus; SiRV, Simian rotavirus; PoRV, Porcine rotavirus; Hu/MuRV, Human/Murine rotavirus.

Sequence comparison of the structural proteins of BAV (VP1, VP2, VP3, VP4, VP8, VP9, and VP10) (Figure 4) with those of other members of the *Reoviridae* have shown that VP9 and VP10 of BAV have similarities to VP8 and VP5 subunits of the outer coat protein VP4 of rotavirus A (*37*). This finding was further confirmed when the crystal structure of BAV VP9 was determined and showed structural similarities to rotavirus VP8 (*38*). In addition, VP3 of BAV, which is the guanylyltransferase of the virus (*39*), exhibited significant amino acid identity with the VP3 of rotavirus, which is also a guanylyltransferase. These data suggest an evolutionary relationship between rotaviruses and seadornaviruses (Figure 5).

### Clinical Features

The only seadornavirus isolated from humans and associated with human disease is BAV. Persons infected with BAV exhibited flulike symptoms, myalgia, arthralgia, fever, and encephalitis (*33*). A serologic diagnostic assay was developed (*40*) based on VP9, the outer coat protein responsible for cell attachment and neutralization. Patients infected with BAV have shown a 4-fold increase in anti-BAV antibody titers in paired serum specimens tested by ELISA, showing an immune response to the virus infection (*34*). Molecular diagnostic assays have also been designed for detecting BAV and KDV (*36*). RT-PCR assays were validated in infected murine model, in which the genome could be detected as early as 3 days postinfection. PCR assays identify differences between genotypes A and B of BAV based on the length of the amplicon obtained by specific primers in segment 9. A PCR assay was recently developed for LNV based on sequence of segment 12 that allows detection of the genome in infected mouse blood (H. Attoui, unpub. data).

### Treatment and Immunity

No specific treatment exists for infections caused by any members of the family *Reoviridae*. In infections with CTFV or BAV, symptomatic treatment includes acetaminophen for relief of fever and pain. Patients infected with CTFV show long-lasting immunity. An experimental vaccine was developed in the 1960s and produced long-lasting immunity, but production was stopped in the 1970s. Patients infected with BAV show a strong immunologic response (*35*). Mice experimentally infected with BAV develop viremia. Clearance of the virus from the circulation occurs concomitantly with the appearance of anti-BAV antibodies (*36**,**40*).
